# Clinical features of children with polycythemia vera, essential thrombocythemia, and primary myelofibrosis in Japan: A retrospective nationwide survey

**DOI:** 10.1002/jha2.39

**Published:** 2020-06-27

**Authors:** Hisashi Ishida, Yuji Miyajima, Nobuyuki Hyakuna, Satoru Hamada, Takeo Sarashina, Risa Matsumura, Katsutsugu Umeda, Tetsuo Mitsui, Naoto Fujita, Daisuke Tomizawa, Kevin Y. Urayama, Yasushi Ishida, Takashi Taga, Masatoshi Takagi, Souichi Adachi, Atsushi Manabe, Toshihiko Imamura, Katsuyoshi Koh, Akira Shimada, Leukemia/Lymphoma Committee of the Japanese Society of Pediatric Hematology Oncology (JSPHO)

**Affiliations:** ^1^ Department of Pediatrics Okayama University Hospital Okayama Japan; ^2^ Department of Pediatrics Anjo Kosei Hospital Anjo Japan; ^3^ Department of Pediatrics University of the Ryukyus Hospital Nishihara Japan; ^4^ Department of Pediatrics Asahikawa Medical University Asahikawa Japan; ^5^ Department of Pediatrics Hiroshima University Hospital Hiroshima Japan; ^6^ Department of Pediatrics Graduate School of Medicine Kyoto University Kyoto Japan; ^7^ Department of Pediatrics Yamagata University Hospital Yamagata Japan; ^8^ Department of Pediatrics Hiroshima Red Cross Hospital and Atomic‐bomb Survivors Hospital Hiroshima Japan; ^9^ Children's Cancer Centre National Centre for Child Health and Development Tokyo Japan; ^10^ Department of Social Medicine National Centre for Child Health and Development Tokyo Japan; ^11^ Graduate School of Public Health St. Luke's International University Tokyo Japan; ^12^ Pediatric Medical Centre Ehime Prefectural Central Hospital Matsuyama Japan; ^13^ Department of Pediatrics Shiga University of Medical Science Otsu Japan; ^14^ Department of Pediatrics and Developmental Biology Tokyo Medical and Dental University Tokyo Japan; ^15^ Department of Human Health Science Kyoto University Kyoto Japan; ^16^ Department of Pediatrics Hokkaido University Hospital Sapporo Japan; ^17^ Department of Pediatrics Graduate School of Medical Science Kyoto Prefectural University of Medicine Kyoto Japan; ^18^ Department of Hematology/Oncology Saitama Children's Medical Centre Saitama Japan

**Keywords:** essential thrombocythemia, myeloproliferative neoplasm, pediatric, polycythemia vera, primary myelofibrosis

## Abstract

**Background:**

Philadelphia‐negative (Ph‐negative) myeloproliferative neoplasms (MPNs), including polycythemia vera (PV), essential thrombocythemia (ET), and primary myelofibrosis (PMF), are exceptionally rare during childhood. Thus, clinical features of pediatric Ph‐negative MPNs remain largely unknown. This study was therefore performed to address this.

**Methods:**

We performed a retrospective study to collect clinical information of children diagnosed with Ph‐negative MPNs from 2000 to 2016 using questionnaires in qualified institutions in Japan. The results obtained from the questionnaire survey were then combined with those from the national registry data.

**Results:**

Among 50 children identified, five had PV, 44 had ET, and one had PMF. Median age at diagnosis was 14.0, 9.0, and 0 years, respectively. Male to female ratio was 4:1, 21:23, and 1:0, respectively. Detection rates of the *JAK2* V617F variant were 0/5 in PV and 9/39 in ET. Frequencies of complications, such as thrombosis and subsequent leukemia, were lower than complication frequencies in adults. We identified two children who developed subsequent leukemia, which has not been reported previously, and one of them died.

**Conclusion:**

This is the first nationally representative survey of pediatric Ph‐negative MPNs. Given its rarity, an international collaboration with comprehensive genetic analyses might be needed to fully elucidate the clinical and genetic features.

## 1 INTRODUCTION

Myeloproliferative neoplasms (MPNs) are clonal hematopoietic disorders characterized by the overproduction of differentiated hematopoietic cells. Philadelphia‐negative (Ph‐negative) MPNs include polycythemia vera (PV), essential thrombocythemia (ET), and primary myelofibrosis (PMF). Ph‐negative MPNs are typically found in older adults and are exceptionally rare during childhood [1, 2, 3].

Although hematologic findings are similar between cases of adult and pediatric MPNs in terms of clinical findings and genetic events, considerable differences have been indicated by several studies [3, 4, 5, 6]. The diagnostic criteria for MPNs are defined by clinical features, laboratory findings, histopathology, and genetic analysis, which are basically utilized in the evaluation of adults [7, 8, 9]. Recently, three driver mutations in *JAK2*, *CALR*, and *MPL* were incorporated into the diagnostic criteria, but the prevalence of these mutations in pediatric patients is reported to be much lower than that in adults [6, 10]. Consequently, the diagnosis of childhood MPN often relies on clinical and laboratory findings in combination with the exclusion of secondary thrombosis, rather than using molecular markers. However, given the low incidence of the disease, studies on clinical and laboratory features of children with MPNs on a national scale are scarce.

Prognostic factors also differ between adults and children with MPNs. For instance, older age and past thrombotic events are often regarded as risk factors for thrombosis [11, 12], but children being younger rarely have past histories of thrombotic events. There is currently no consensus on risk factors associated with complications or poor prognoses for children with MPNs, nor on the optimal clinical management of this group of patients [1, 2].

In this article, we present the results of a retrospective survey of pediatric MPNs in Japan that was performed on a national scale. Our objective was to clarify the clinical characteristics, incidence, complications, and types of driver mutations among children with MPNs.

## 2 PATIENTS AND METHODS

We focused on three primary types of Ph‐negative MPNs in this retrospective study—PV, ET, and PMF. Patients with incorrect diagnoses, patients who did not consent to the study, or patients with insufficient clinical information were excluded. The study was conducted as a project of the Japanese Society of Pediatric Hematology/Oncology (JSPHO), Leukemia and Lymphoma Committee. The study protocol was approved by the JSPHO and Okayama University Hospital institutional review boards.

### 2.1 Questionnaire survey

All members of the JSPHO were asked if they were in charge of pediatric or young‐adult patients <30 years of age who had been diagnosed with Ph‐negative MPNs at some point between 2000 and 2016. Diagnoses were based on the 2008 and 2016 World Health Organization (WHO) classification or on physician decisions [9, 13]. Patients with secondary myeloproliferative conditions were excluded by the charge doctors. Responses to the questionnaires were received from 136 institutions, including from physicians at all 106 board‐certified educational institutes of JSPHO. The clinical information of those patients was then collected by administering a questionnaire to the physicians who saw the relevant patients. The follow‐up questionnaire requested specific patient information, including age at diagnosis, sex, date of diagnosis, date of birth, family history of MPN, results of complete blood counts and bone marrow morphology at diagnosis, results of karyotype and genetic analyses, disease complications, and details regarding treatments and outcomes. Patients who did not consent to this questionnaire survey were excluded from further analyses.

### 2.2 JSPHO registry survey

In addition to the questionnaires, we also referred to the JSPHO national registry [14], and the data of patients diagnosed with Ph‐negative MPNs during the 2000‐2016 period were extracted. Patient information from the registry, such as date of birth and patient initials, was used to identify duplications with the data from the questionnaire surveys. For nonduplicated patients, their clinical information was collected by sending the same questionnaire to the physicians in charge of these patients. Patients who did not consent to this questionnaire survey were excluded from further analyses. The national registry data included diagnosed patients who were <20 years of age.

## 3 RESULTS

### 3.1 Overview

**FIGURE 1:**
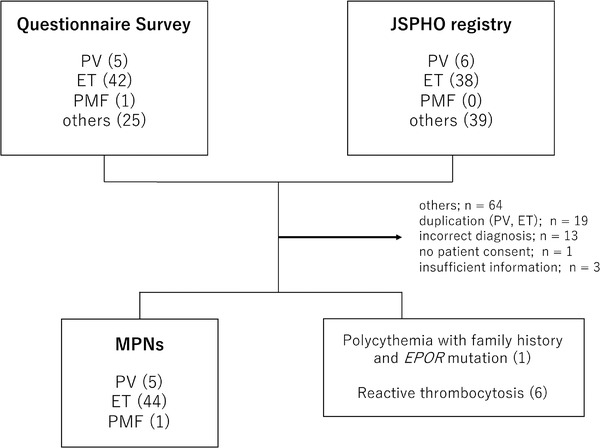
Schematic of the current retrospective study including the numbers of eligible patients Abbreviations: ET, essential thrombocythemia; JSPHO, Japanese Society of Pediatric Hematology/Oncology; MPNs, myeloproliferative neoplasms; PMF, primary myelofibrosis; PV, polycythemia vera.

Responses to the questionnaires were received from 136 institutions, including all 106 board‐certified educational institutes of JSPHO. A total of 73 patients with Ph‐negative MPNs from 44 institutions were reported. From the data available in the JSPHO national registry, 83 patients with Ph‐negative MPNs were identified. The data from these two cohorts were combined and the duplicate cases or those with inadequate information were excluded. One patient who did not consent to this study was excluded from further analyses. Ultimately, 57 cases were subjected to further analysis (Figure 1). Cases classified as “others” included mastocytosis (n = 26), chronic eosinophilic leukemia (n = 6), chronic myelomonocytic leukemia (n = 4), atypical chronic myeloid leukemia, myelodysplastic syndrome/MPN unclassifiable (n = 17), and patients with insufficient data (n = 5).

One patient who experienced polycythemia with a family history and germline *EPOR* mutation was also excluded. Among the patients who were reported to be ET patients, platelet numbers decreased to normal values (<450 × 10^9^/L) during the follow‐up period in six cases. Therefore, these patients were thought to have reactive thrombocytosis.

Of the patients ultimately included in the study, five were diagnosed with PV, 44 were diagnosed with ET, and one was diagnosed with PMF. One patient with PV and one patient with ET of the 50 total patients included (4.0%) developed subsequent leukemia. One patient with PV died of subsequent acute myeloid leukemia with the remaining 49 patients being alive at their last follow‐up. Median follow‐up duration of all included patients with MPNs was 54.4 months (0.2‐237.1 months). The clinical characteristics of the patients included in the study are summarized in Table 1. As only one patient was diagnosed with PMF during the study period, this patient is discussed in a separate section.

**TABLE 1:** The clinical characteristics of the patients with MPNs in the study

	PV (n = 5)	ET (n = 44)	PMF (n = 1)
Sex (%)			
Male	4 (80.0)	21 (47.7)	1 (100)
Female	1 (20.0)	23 (52.3)	0 (0)
Age at diagnosis	14.0 [1.0, 15.0]	9.0 [0.0, 15.0]	0
White blood cells (× 10^9^/L)	8.0 [4.5, 15.7]	11.5 [5.5, 21.5]	18
Hemoglobin (g/L)	193 [163, 245]	125 [88, 165]	9.0
Platelets (× 10^9^/L)	178 [137, 217]	1432 [688, 2580]	20
Family history (%)	1 (20.0)	3 (6.8)	0 (0)
Hemorrhage (%)	0 (0)	4 (9.1)	0 (0)
Thrombosis (%)	0 (0)	3 (6.8)	0 (0)
Organ failure (%)	1 (20.0)	0 (0)	0 (0)
Subsequent leukemia (%)	1 (20.0)	1 (2.3)	0 (0)
Subsequent malignancy other than leukemia (%)	0 (0)	0 (0)	0 (0)
Myelofibrosis (%)	1 (20.0)	3 (6.8)	1 (100)
*JAK*2 V617F mutation (%)	0/5 (0)	9/39 (23)	Not tested
*CALR* mutation (%)	Not tested	1/12 (8)	Not tested
*MPL* mutation (%)	Not tested	0/15 (0)	Not tested
Median follow‐up duration (months)	132.2 (21.8‐237.1)	46.5 (0.2‐174.2)	147.1

Abbreviations: ET, essential thrombocythemia; MPNs, myeloproliferative neoplasms; PMF, primary myelofibrosis; PV, polycythemia vera.

### 3.2 Clinical characteristics

There was a predominance of males among the patients with PV and a slight predominance of females among patients with ET. Median age at diagnosis was 14.0 years old for patients with PV and 9 years old for patients with ET.

### 3.3 Biological characteristics

Results of blood analyses at presentation are presented in Table 1. For patients with PV, the median leukocyte count was 8.0 × 10^9^/L, hemoglobin concentration was 193 g/L, and platelet count was 178 × 10^9^/L. For patients with ET, the median leukocyte count was 11.5 × 10^9^/L, hemoglobin concentration was 125 g/L, and platelet count was 1432 × 10^9^/L.

### 3.4 Chromosomal and genetic analyses

Karyotype analyses were performed for five patients with PV and 41 patients with ET. Only one patient with PV demonstrated a karyotype abnormality [46, XX, t(1;1)(q34;q21)]. This patient subsequently developed acute myeloid leukemia (see “Complications” below).

Results of the genetic analyses are presented in Table 1. All five patients with PV were negative for the *JAK2* V617F variant, and these patients were not analyzed for *JAK2* exon 12 mutations. One patient had a family history of erythrocytosis. Of the patients tested, the *JAK2* V617F variant was present in nine of 39 patients of the patients with ET. Of the patients with ET that were tested, the *CALR* mutation was detected in one of 12 patients, whereas the *MPL* variant was not detected in any of the 15 patients analyzed.

### 3.5 Complications

No patient with PV experienced a hemorrhagic or thrombotic event. Of the five patients with PV, three received phlebotomy and three received no therapy during the follow‐up period. One patient who developed subsequent leukemia had received hydroxyurea prior to leukemia development (Table 2).

**TABLE 2:** Treatments of patients with PV, ET, and reactive thrombocytosis

	PV	ET	Reactive thrombocytosis
	(n = 5)	(n = 44)	(n = 6)
No therapy	3 (60%)	11 (25%)	2 (33%)
Phlebotomy	2 (40%)	0 (0%)	0 (0%)
Aspirin	0 (0%)	21 (48%)	4 (67%)
Dipyridamole	0 (0%)	1 (2%)	0 (0%)
Ticlopidine	0 (0%)	1 (2%)	0 (0%)
Anagrelide	0 (0%)	12 (27%)	0 (0%)
Hydroxyurea	1 (20%)	6 (14%)	0 (0%)

Abbreviations: ET, essential thrombocythemia; PV, polycythemia vera.

Four patients with ET experienced hemorrhage and three patients with ET experienced thrombotic events. Several patients with ET required additional therapies, such as anagrelide or hydroxyurea (Table 2).

**FIGURE 2:**
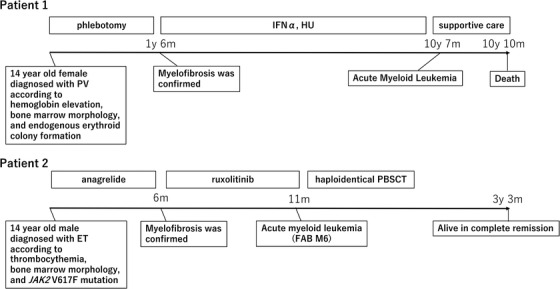
Clinical course of two patients with pediatric MPNs who developed subsequent leukemia. Each number over the arrow indicates the time after the diagnosis of their MPNs Abbreviations: ET, essential thrombocythemia; FAB, French‐American‐British; HU, hydroxyurea; IFN, interferon; PV, polycythemia vera.

Two patients who developed myelofibrosis and subsequent acute myeloid leukemia were identified, one patient with PV and one with ET. The clinical courses of these two patients are summarized in Figure 2. Briefly, Patient 1 was a 14‐year‐old girl diagnosed with PV according to elevated hemoglobin (165 g/L), bone marrow findings, and endogenous erythroid colony formation in vitro. She had a karyotype abnormality at diagnosis [46, XX, t(1;1)(q34;q21)]. Although initially controlled with a phlebotomy, she developed myelofibrosis 18 months after the initial diagnosis. She then received IFN‐α and subsequently hydroxyurea. Her disease progressed to acute myeloid leukemia 10 years after the initial diagnosis. Ultimately, she died of leukemia.

Patient 2 was a 14‐year‐old boy diagnosed with ET according to an elevated platelet count (10,100 × 10^9^/L), bone marrow findings, and the detection of *JAK2* V617F variant. He had no karyotype abnormality. Initially, he was treated with anagrelide. He experienced myelofibrosis 6 months after the initial diagnosis and was then prescribed ruxolitinib. Acute myeloid leukemia developed 5 months after the development of myelofibrosis. He received an human leukocyte antigen‐haploidentical peripheral blood stem cell transplantation and was alive and in complete remission at last follow‐up.

### 3.6 Primary myelofibrosis

Only one patient was diagnosed with PMF. The patient was male and at diagnosis was 11 months of age. He presented with no hemorrhage or thrombosis. This patient received immunosuppressive therapy followed by a stem cell transplantation. He was still alive at his most recent follow‐up, 12 years after the initial diagnosis.

### 3.7 Comparison between ET and reactive thrombocytosis

Among cases who were reported to be ET patients, platelet numbers decreased to normal values (<450 × 10^9^/L) during the follow‐up period in six cases. These patients were thought to have reactive thrombocytosis, and thus were excluded from the MPN group. Here, we compared the clinical characteristics between ET patients and these patients with reactive thrombocytosis. The median age at diagnosis of patients with reactive thrombocytosis (7.5 years old) was less than that of patients with ET (9 years old). For blood analyses, similar results were obtained for patients with reactive thrombocytosis and patients with ET. One patient with reactive thrombocytosis showed 46, XY, inv(9)(p11q13), which was regarded as a normal variant [15]. The *JAK2* V617F variant was present in none of the seven patients with reactive thrombocytosis. No patient was tested for *CALR* or *MPL* mutations. No patients with reactive thrombocytosis experienced either hemorrhage or thrombosis, and the majority of these patients were controlled with aspirin or observation only (Table 2).

## 4 DISCUSSION

To the best of our knowledge, this is the first nationally representative survey aimed at clarifying the clinical aspects of pediatric Ph‐negative MPNs. We initially investigated the characteristics of children with Ph‐negative MPNs in Japan using a questionnaire survey. Responses to the questionnaires were received from all 106 board‐certified educational institutes of JSPHO. Next, in an attempt to capture additional cases, we supplemented our approach by accessing data from the JSPHO national registry. The results from the two methods were combined, allowing us to perform the comprehensive survey reported.

Males predominated the patients with PV, whereas there was a slight female predominance in patients with ET, which was consistent with previous reports of large studies [3, 4]. Median ages of these patients were also similar to the results of a recent systematic review [3]. Therefore, the biological characteristics of the patients included in our current study appear to be compatible with those of patients included in previous reports.

Blood analysis results at the time of diagnosis for the patients with PV in our current study differed from those of the previous systematic review; however, they were similar to those of a large Italian study [3, 4]. According to a systematic review [3], the prevalence of *JAK2* V617F mutation was only 24% among children with PV, which is much lower than that of adult patients [3, 6, 7, 10]. Considering this number, we think that it is not surprising that our five PV patients did not have *JAK2* V617F mutation. However, definitive conclusions cannot be drawn due to limitation in patient sample size.

In the current study, platelet numbers decreased to normal values during the follow‐up period in six cases of thrombocythemia. These patients were thought to have reactive thrombocytosis, and thus were excluded from the MPN group. When we compared these patients with patients with ET, the blood analysis results at diagnosis appeared similar for the patients with ET, those with reactive thrombocytosis, and the patients included in the systematic review [3]. In addition, for patients with ET, frequencies of three driver mutations in our study were similar to the results reported in the systematic review [3]: specifically, *JAK2* V617F 28% versus 31%, *CALR* 8% versus 10%, and *MPL* 0% versus 2%, respectively. However, the prevalence of all three major driver mutations was much lower in the pediatric population than in adults [16, 17], which was consistent with previous reports [5, 6, 10, 18–21]. As patients with ET do not have distinctive features based on blood analysis and because the majority of these patients lack any of the three driver mutations, it is often difficult at the time of diagnosis to differentiate true patients with ET from patients with reactive thrombocytosis. This is further supported by several reports that have demonstrated the difficulty in excluding cases of secondary thrombocytosis from cases of true ET [8, 22, 23]. On the other hand, these two groups were different in terms of complications, as patients with reactive thrombocytosis experienced no complications after a median 82.3‐month follow‐up.

The frequency of complications, such as thrombosis, hemorrhage, transformation to myelofibrosis, and development of leukemia, was much lower in our evaluated pediatric patients than in adult patients [16, 17]. This finding was also consistent with previous reports regarding pediatric patients [3, 4]. Therefore, although our study included relatively short durations of follow‐up, the prognosis of pediatric patients with Ph‐negative MPNs appeared to be comparatively good.

Transformation to myelofibrosis and/or leukemia is a severe complication in adult patients with MPNs, but the incidence is much lower in children with MPNs [3, 4]. Notably, we identified two patients in our study population who developed myelofibrosis and subsequent acute myeloid leukemia, one patient with PV and one with ET (Figure 2). These two patients were relatively old for our cohort (14 years old at diagnosis) and both developed myelofibrosis before leukemic transformation.

Several risk factors associated with leukemia development in patients with PV have been reported, including age >61 years old, leukocyte counts >15 × 10^9^ /L, and an abnormal karyotype [24, 25, 26]. With respect to our patient with PV, her initial leukocyte count and abnormal karyotype were considered to be risk factors. It is noteworthy that she was the only patient in our cohort who had an abnormal karyotype. She had chromosome 1 abnormalities, which have been reported to be most frequently detected among patients with Ph‐negative MPNs [27].

No pediatric patients with Ph‐negative MPNs described in the previous systematic review developed leukemia [3]. Therefore, our cases provide important information toward our understanding of childhood Ph‐negative MPNs.

Information regarding the treatment of children with MPNs has also been scarce. European LeukemiaNet recommendations are often used in the management of MPNs, but these are not applied to pediatric MPNs [12]. Our data indicate that there are considerable differences between adult and pediatric MPNs in terms of clinical characteristics, incidences, types of driver mutations, and complications. Therefore, specialized diagnostic criteria and clinical management standards for children with Ph‐negative MPNs should be established.

Our study had several limitations. First, we originally aimed to collect data of patients under 30 years of age; however, because we sent the questionnaires to pediatricians who usually see only children, all the patients included in our study were 15‐year old or younger. As the incidence of all types of MPNs increases with age [28], the characteristics of adolescent and young adults with MPNs remain a matter needing further evaluation. Second, genetic testing of patients with MPNs in Japan is not centralized. As a result, there were several patients included in our study that did not receive sufficient genetic analysis. Consequently, we were unable to determine the precise genetic profile of children with MPNs. Comprehensive and centralized genetic analyses of children with MPNs should be performed in the future.

In summary, our current data revealed the clinical characteristics of children with MPNs and suggested that complications for children with MPNs are less frequent than those for adult patients. Therefore, the prognosis of children with MPNs is relatively good. However, it should be kept in mind that some children with MPNs may develop myelofibrosis and subsequent leukemia. Finally, given the rarity of pediatric Ph‐negative MPNs, international collaborations including comprehensive genetic analyses might be needed to fully elucidate the clinical and genetic features of these hematopoietic disorders.

## AUTHOR CONTRIBUTIONS

HI and AS designed the study, performed the evaluation, and wrote the paper. YM, NH, and SH provided patient information and important clinical data. All authors revised the manuscript for important intellectual content and approved the final version of the manuscript.

## CONFLICT OF INTEREST

The authors declare no conflict of interest.
